# Transcriptome analysis of radish sprouts hypocotyls reveals the regulatory role of hydrogen-rich water in anthocyanin biosynthesis under UV-A

**DOI:** 10.1186/s12870-018-1449-4

**Published:** 2018-10-11

**Authors:** Xiaoyan Zhang, Nana Su, Li Jia, Jiyuan Tian, Han Li, Lisha Huang, Zhenguo Shen, Jin Cui

**Affiliations:** 1 College of Life Sciences, Nanjing Agricultural University, Nanjing, China; 2 Biomics Biotech Co. Ltd, Beijing, China

**Keywords:** Anthocyanin, Molecular hydrogen, Radish sprouts, Transcriptome, UV-A

## Abstract

**Background:**

Hydrogen gas (H_2_) is the most abundant element in the universe, and has been reported to act as a novel beneficial gaseous molecule in plant adaptive responses. Radish sprouts are popular because they contain substantial amounts of antioxidants and health-promoting compounds, such as anthocyanin and glucosinolates. Although radish sprouts accumulated more anthocyanin under UV-A after treatment with hydrogen-rich water (HRW), the molecular mechanism responsible is still elusive. To explore these mechanisms, RNA-seq analysis was used.

**Results:**

Four cDNA libraries from radish sprout hypocotyls were constructed, and a total of 14,564 differentially expressed genes (DEGs) were identified through pairwise comparisons. By Gene Ontology (GO) and Kyoto Encyclopedia of Genes and Genomes (KEGG) analysis, these unigenes were found to be implicated in light signal perception and transduction, starch and sucrose metabolism, photosynthesis, nitrogen metabolism and biosynthesis of secondary metabolites. The MYB-bHLH-WD40 complex accounted for the majority of the transcription factors found to be involved in anthocyanin biosynthesis, and levels of transcripts for this complex were in accordance with the anthocyanin concentrations observed. In addition, other transcription factors (such as NAC, bZIP and TCP) might participate in HRW-promoted anthocyanin biosynthesis. Furthermore, the signaling processes of plant hormones, MAPKs and Ca^2+^ might be involved in HRW-promoted anthocyanin biosynthesis under UV-A. The expression patterns of 16 selected genes were confirmed using qRT-PCR analysis.

**Conclusions:**

Taken together, the results of this study may expand our understanding of HRW-promoted anthocyanin accumulation under UV-A in radish sprouts.

**Electronic supplementary material:**

The online version of this article (10.1186/s12870-018-1449-4) contains supplementary material, which is available to authorized users.

## Background

Radish (*Raphanus sativus* L.) sprouts are commonly consumed due to their potential benefits for human health. The bioactive phytochemicals in radish sprouts include phenolics, vitamin C, chlorophylls, carotenoids and glucosinolates [1, 2]. Color is one of the most striking features of vegetables, and red-skinned radish cultivars, which are rich in anthocyanins, can attract consumers with their lovely appearance. Anthocyanins are important phenolic compounds responsible for plant organ colors such as blue, purple and red [3]. Apart from the well-known physiological function of attracting pollinators, anthocyanins also protect plants from a variety of biotic and abiotic stresses, such as bacterial and fungal infections, herbivory, UV radiation, temperature extremes, drought and nitrogen deficiency [4, 5]. In addition, there is mounting evidence indicating that anthocyanins act as antioxidants to protect humans from cardiovascular disease, cancer, inflammation and other chronic diseases [6–8]. Thus, an increasing amount of attention has been paid to the development of anthocyanin-rich diets.

Anthocyanins are biosynthesized via the flavonoid pathway, which is controlled by two types of genes: structural genes and regulatory genes. Structural genes encode the enzymes necessary for anthocyanin biosynthesis and can be divided into the early biosynthetic genes (EBGs) and the late biosynthetic genes (LBGs) [9]. The enzyme products of the EBGs, including PAL, C4H, 4CL, CHS, CHI, and F3H, carry out the early steps of anthocyanin biosynthesis to the formation of dihydroflavonols. The enzyme products of the LBGs, including F3’H, DFR, ANS, LDOX and UFGT, lead to the biosynthesis of anthocyanins [9, 10]. The regulatory genes, which regulate anthocyanin biosynthesis at the transcriptional level, encode transcription factors (TFs). Three types of transcription factors involved in anthocyanin biosynthesis have been reported: WD-repeat proteins and bHLH and MYB transcription factors [11, 12]. Together, these factors form a ternary complex, the MYB–bHLH–WD repeat (MBW) complex, to orchestrate anthocyanin biosynthesis. Numerous factors have been reported to regulate the biosynthesis of anthocyanins, such as light, temperature, nitrogen depletion, salt stress, sucrose and plant hormones [13–16]. Previous studies have demonstrated that anthocyanin accumulation can be induced by exposure to ultraviolet (UV) light, but most of those studies focused on UV-B-induced anthocyanin biosynthesis. UV-A (320–400 nm), which is in the range of long-wave UV and has lower energy than UV-B, was also reported to induce anthocyanin biosynthesis in *Arabidopsis* [17], turnips [18, 19], tomatoes [20], and blueberries [21]. It is generally recognized that UV-A plays a key role in activating the genes responsible for anthocyanin biosynthesis [18].

Recently, H_2_ has attracted attention as a selective antioxidant and signal molecule [22, 23]. Studies in animals indicated that H_2_ acted as a novel antioxidant that selectively reduced the levels of toxic reactive oxygen species [24] and exerted anti-apoptotic, anti-allergy and anti-inflammatory effects [25–27]. A growing body of evidence indicates that H_2_ plays an important role in plants, especially in stress defence [28–31]. For example, when H_2_ was supplied in the form of hydrogen-rich water (HRW), it could alleviate calcium- and salt-induced oxidative stress in rice and Chinese cabbage, respectively [32, 33]. A similar protective effect was found in alfalfa, showing that HRW conferred tolerance to UV-B-induced oxidative damage partially by the manipulation of (iso)flavonoid metabolism and antioxidant defense [34]. In another case, H_2_ was reported to regulate cucumber adventitious root development [35]. Our previous study showed that UV-A-induced toxicity was alleviated by HRW via the re-establishment of ROS homeostasis and the promotion of anthocyanin synthesis in radish sprouts [36]. However, the regulatory role of H_2_ in anthocyanin metabolism under UV-A radiation has not been thoroughly studied in radish sprouts.

In this study, we used RNA-seq analysis to understand the mechanisms of H_2_-promoted anthocyanin biosynthesis under UV-A radiation in radish sprout hypocotyls. Thus, based on the analysis of DEGs found by pairwise comparisons, a model regulatory network for H_2_-promoted anthocyanin biosynthesis in radish sprouts was proposed. The results of our study provide an overview of transcriptomic responses to H_2_ in radish and thus extend our understanding of the biological functions of H_2_ in plants.

## Methods

### Plant materials and culture conditions

Radish (*Raphanus sativus* L., cultivar ‘Yanghua’) sprouts were cultured in nutrient medium (1/4 Hoagland’s solution prepared with distilled water or HRW) in the dark for 1 day at 25 °C and then transferred to incubators with white light (W) or UV-A illumination (Ningbo Saifu Instrument Experimental Factory, Zhejiang, China). The light intensity of the white light was 30 ± 5 μmol m^− 2^ s^− 1^. The UV-A radiation was 5.5 W m^− 2^, as measured by a portable digital radiometer for UV (UV-340A, Lutron, Taiwan). The temperature and relative humidity in the growth chamber were 25 °C and 80%, respectively. The nutrient medium was replaced every 12 h. The total anthocyanin contents were measured after 12, 24 and 36 h of light treatment. Hypocotyl tissues were sampled immediately after 24 h light treatment, frozen in liquid nitrogen, and stored at − 80 °C for further analysis.

### Preparation of HRW

Purified H_2_ gas (99.99%, *v*/v) was produced with a hydrogen gas generator (SHC-300, Saikesaisi Hydrogen Energy Co., Ltd., Jinan, China). In our experimental conditions, the H_2_ concentration in freshly prepared HRW (100% saturation) analyzed by gas chromatography was 781 μmol L^− 1^ [34]. Endogenous H_2_ production was measured by gas chromatography (Agilent 7890A, Wilmington, DE, USA) with a thermal conductivity detector [28].

### Determination of endogenous H_2_ concentrations

Endogenous H_2_ concentration was measured by gas chromatography (GC) (Agilent 7890A) [31].

### Determination of anthocyanin concentration

Anthocyanin concentrations in hypocotyls of radish sprouts were measured in accordance with previously described methods [37]. Briefly, 0.5 g of freeze-dried hypocotyl samples were extracted in 5 mL of 0.1% HCl/methanol. (A_530_–0.25 × A_657_) g^− 1^ dry weight (DW) was used to quantify the relative amount of anthocyanin.

### RNA extraction, library construction, and transcriptome sequencing

Total RNA for RNA-seq was extracted from hypocotyls using Trizol reagent (Invitrogen, Carlsbad, CA, USA). The purity and concentration of RNA were checked using the NanoPhotometer® spectrophotometer (IMPLEN, CA, USA) and the Qubit® RNA Assay Kit in a Qubit® 2.0 Fluorometer (Life Technologies, CA, USA), respectively. In addition, the integrity of RNA was assessed using the RNA Nano 6000 Assay Kit of the Bioanalyzer 2100 system (Agilent Technologies, CA, USA).

Total RNA from each sample was used for Illumina sequencing at Novogene Bioinformatics Technology Co. Ltd. (Beijing, China). Sequencing libraries were generated using NEBNext® Ultra™ RNA Library Prep Kit for Illumina® (NEB, USA) according the manufacturer’s instructions, and index codes were added to attribute sequences to each sample.

### Quantification of gene expression levels

HTSeq v0.6.1 was used to count the number of reads mapped to each gene. In addition, the FPKM (fragments per kilobase of exon model per million mapped reads) of each gene was calculated based on the length of the gene and the number of reads mapped to the gene [38].

### Differential expression analysis

Differential expression analysis of pairs of groups was performed using the DESeq R package (1.18.0). The *P*-values were adjusted using Benjamini and Hochberg’s approach for controlling the false discovery rate. Genes with an adjusted P-value < 0.05 found by DESeq were assigned as differentially expressed [39, 40].

### GO and KEGG enrichment analysis of DEGs

Gene Ontology (GO) enrichment analysis of DEGs was implemented by the GOseq R package. GO terms with corrected P-values < 0.05 were considered to be significantly enriched by differentially expressed genes [41]. KOBAS software was used to test the statistical enrichment of DEGs in KEGG pathways.

### Quantitative PCR analysis

The transcription levels of 16 genes involved in anthocyanin biosynthesis pathway were analyzed using quantitative real-time PCR (qRT-PCR). Total RNA was extracted from radish hypocotyls using Trizol reagent (Invitrogen, USA) and 1 μg aliquots were treated with RNase-free DNase I to remove genomic DNA and then reverse transcribed using a RevertAid First Strand cDNA Synthesis Kit (Thermo Scientific, USA). qRT-PCR was carried out on a Mastercycler ep realplex Real-time PCR System (Eppendorf, Hamburg, Germany) using Bestar SYBR Green qPCR Mastermix (DBI, Bioscience Inc., Germany) [36]. Reactions were performed at 95 °C for 2 min, 40 cycles of 95 °C for 10 s, 60 °C for 30 s and 72 °C for 30 s. Three biological replicates were applied. The specific primers were designed using the Primer Premier 6.0 software. The primer sequences were listed in Additional file 1: Table S1 in the supporting information. *Actin2* and *EF1* were used as the reference genes [42]. The relative expression levels of the target genes were calculated using the 2^−△△CT^ approach, with normalization of data to the geometric average of two reference control genes [43, 44].

### Statistical analysis

Statistical analysis was performed using SPSS statistic 17.0 software. For statistical analysis, Duncan’s multiple test (*P*<0.05) was chosen. Data are means ± SE from at least three independent biological replicates.

## Results

### HRW promoted UV-A-induced anthocyanin accumulation

**Fig. 1:**
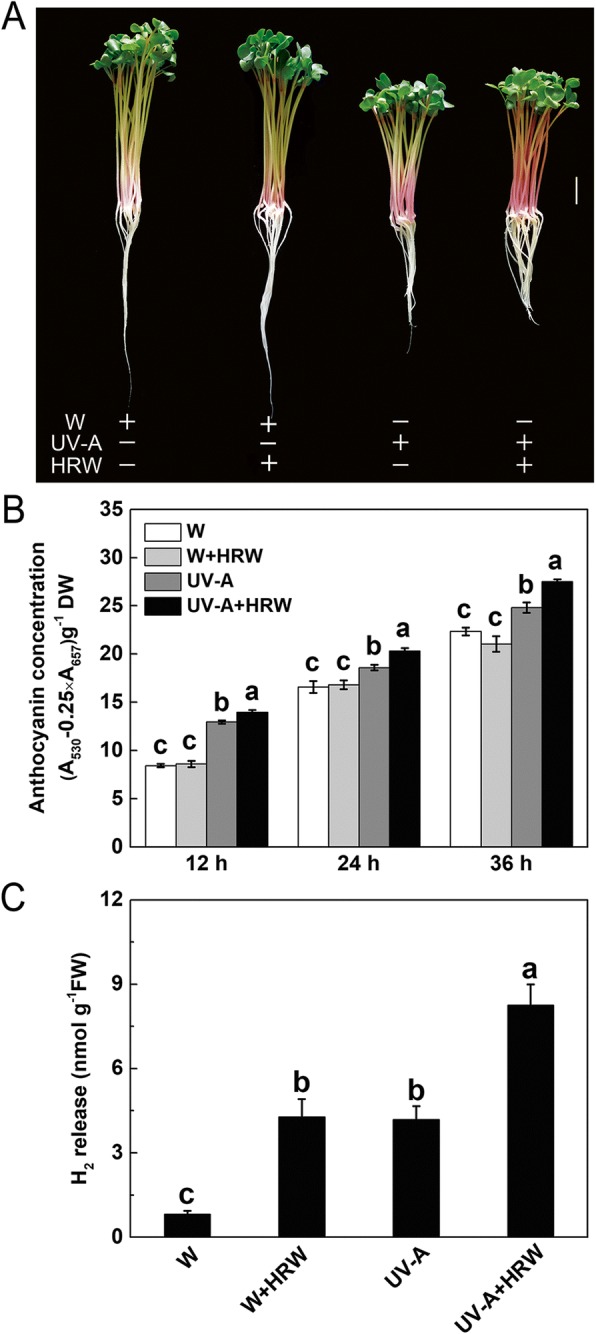
Anthocyanin accumulation and H_2_ release in radish sprouts hypocotyls. **a** The morphology of radish sprouts after 36 h light exposure. Seedlings were cultured under white light (W) or UV-A radiation (UV-A), and the nutrient solution (quarter-strength Hoagland’s solution) were prepared with distilled water or HRW (100% saturation). Scale bar = 1 cm. **b** Total anthocyanin content in the hypocotyls of radish sprouts in different culture time. **c** Hydrogen (H_2_) release in radish sprouts upon UV-A and HRW treatment. Values are the means ± SE of three independent experiments. Bars with different letters are significantly different at *P* < 0.05 according to Duncan’s multiple range test

To assess the effects of HRW on anthocyanin biosynthesis, radish sprouts were exposed to white light (W) or UV-A radiation (UV-A) with and without HRW treatment, and the anthocyanin concentrations were determined after 12, 24 and 36 h of light exposure (Fig. 1). The hypocotyls of radish sprouts grown under UV-A exhibited increased pigmentation, compared with those grown under white light. Moreover, radish sprouts grown under UV-A + HRW showed obviously increased anthocyanin accumulation in hypocotyls comparing with those grown under UV-A (Fig. 1a). As is shown in Fig. 1b, the anthocyanin concentrations of radish sprouts hypocotyls increased in a time-dependent manner. In addition, the anthocyanin concentration under UV-A radiation was significantly higher than that under white light. It is interesting that the anthocyanin concentration under UV-A + HRW treatment was further significantly increased compared with that under UV-A treatment. However, no significant difference was observed between W and W + HRW treatments (Fig. 1b). To investigate whether UV-A affects endogenous H_2_ production, gas chromatography was applied to monitor the production of H_2_. H_2_ concentration was significantly increased in sprouts treatment with UV-A radiation compared to the control (W). Under the same light condition, a significant increase in H_2_ concentration by HRW treatment was observed (Fig. 1c).

To gain insights into the underlying mechanisms of HRW-promoted anthocyanin accumulation, mRNA sequencing of the hypocotyl samples after 24 h of light exposure were conducted.

### Identification of differentially expressed genes

**Fig. 2:**
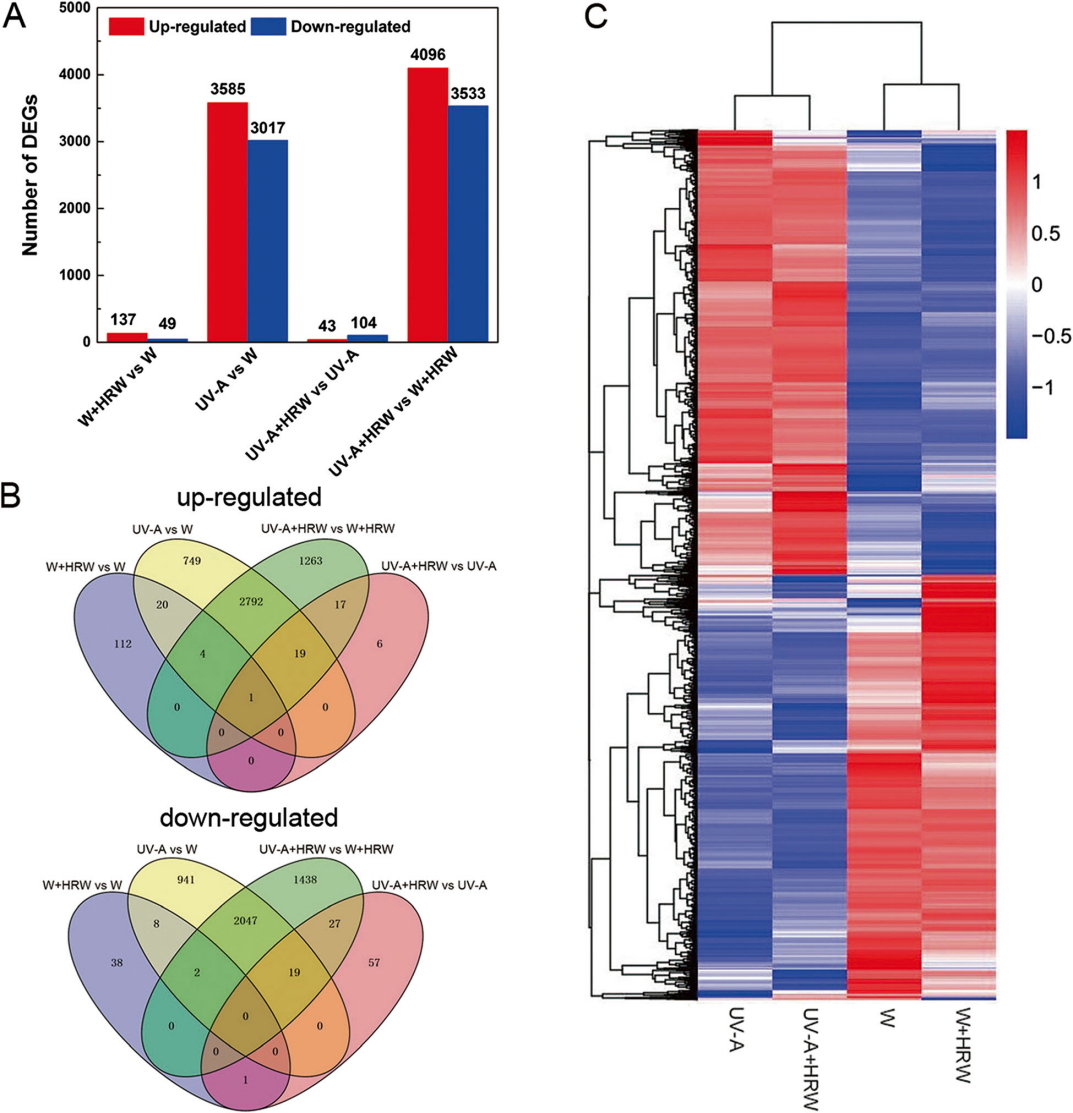
Changes in gene expression profiles of radish sprouts in response to HRW under W and UV-A radiation. **a** Numbers of DEGs in pairwise comparisons of the four treatments; (**b**) Venn diagram showed the number of DEGs in different treatment; (**c**) Heatmap diagrams showed the relative expression levels of total DEGs among the four treatments

Differences in gene expression were examined to analyze the genes that may participate in HRW-promoted anthocyanin biosynthesis under UV-A. A total of 14,564 DEGs in radish sprouts were identified through pairwise comparisons, of which 186, 6602, 147 and 7629 DEGs were found between W + HRW and W, UV-A and W, UV-A + HRW and UV-A, and UV-A + HRW and W + HRW, respectively (Fig. 2a). One hundred thirty-seven genes were up-regulated and 49 genes were down-regulated under the W + HRW treatment compared with the W treatment. Comparison of the W treatment with the UV-A treatment revealed 3585 genes that were up-regulated and 3017 genes that were down-regulated in the latter. Forty-three genes were up-regulated and 104 genes were down-regulated under the UV-A + HRW treatment compared with the UV-A treatment. Twenty of the 3585 up-regulated differentially expressed genes between UV-A and W were also among the genes differentially expressed between UV-A + HRW and UV-A. In addition, 19 of the 3017 down-regulated differentially expressed genes between UV-A and W were also among the genes differentially expressed between UV-A + HRW and UV-A (Fig. 2b). Furthermore, a general overview of the expression pattern was visualized in a heat map (Fig. 2c), which provided an overall understanding of the changes in gene expression. It was shown in Fig. 2c that the expression patterns of most DEGs under W and UV-A treatment were completely opposite, regardless of whether HRW was present or not. Most of the genes with higher expression levels under UV-A had lower expression levels under W, and vice versa. Besides, compared with that of UV-A, most of the DEGs displayed great differences in expression profile under UV-A + HRW treatment.

### Functional classification of the DEGs by GO and KEGG pathway analysis

**Fig. 3:**
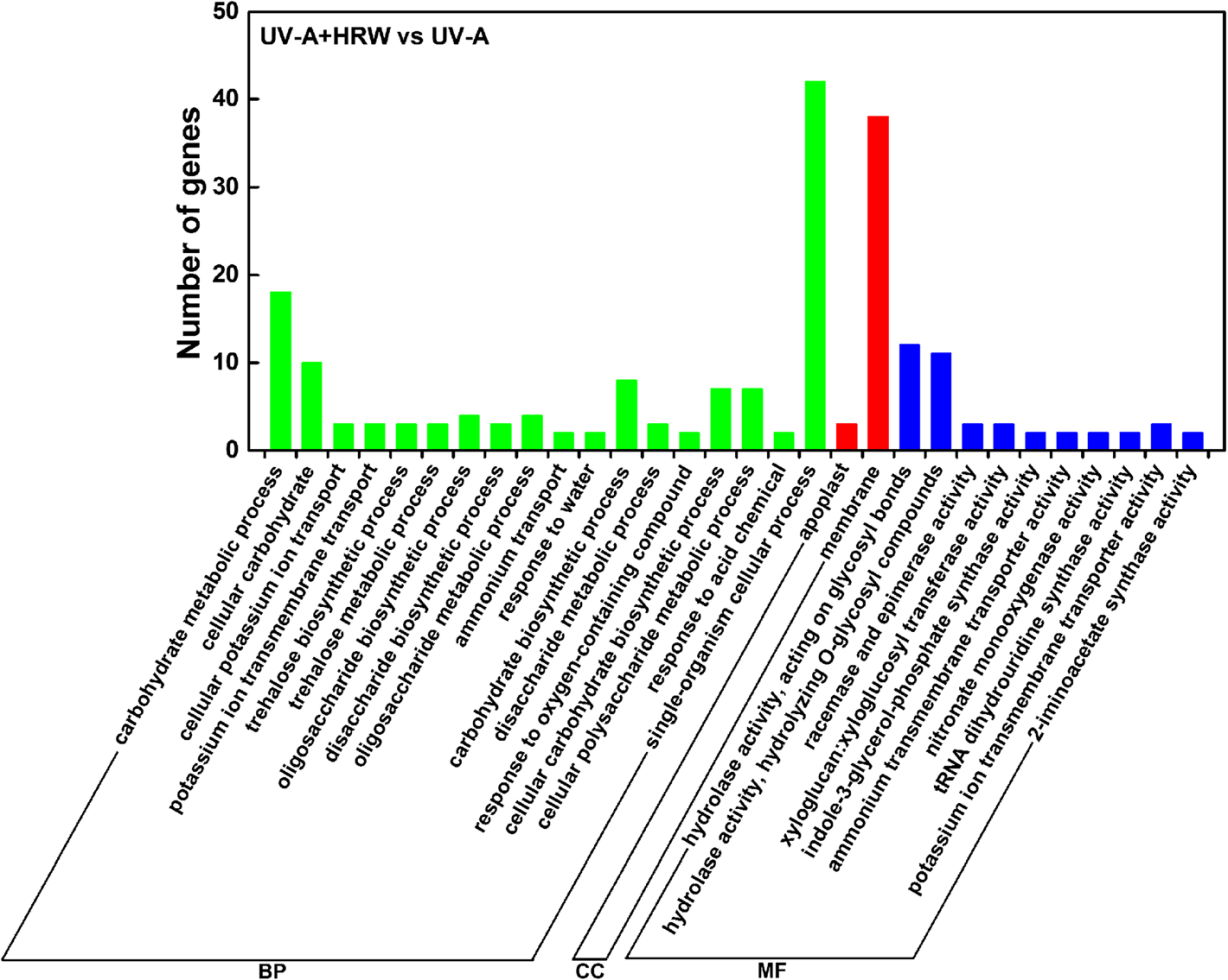
GO analysis of DEGs between UV-A + HRW and UV-A treatments in three main categories. BP: biological process, CC: cellular component, MF: molecular function

To identify the HRW-induced genes under UV-A irradiation, the DEGs were evaluated with GO and KEGG pathway analyses. The proportions of enriched genes in the UV-A + HRW vs UV-A comparison were summarized in three main GO categories (Fig. 3). In the biological process category, the GO terms significantly enriched in the UV-A + HRW vs UV-A comparison included “carbohydrate metabolic process” and “single-organism cellular process”. In the cellular component category, “membrane” was significantly enriched. In the molecular function category, “hydrolase activity, acting on glycosyl bonds” and “hydrolase activity, hydrolyzing O-glycosyl compounds” were significantly enriched.

**Fig. 4:**
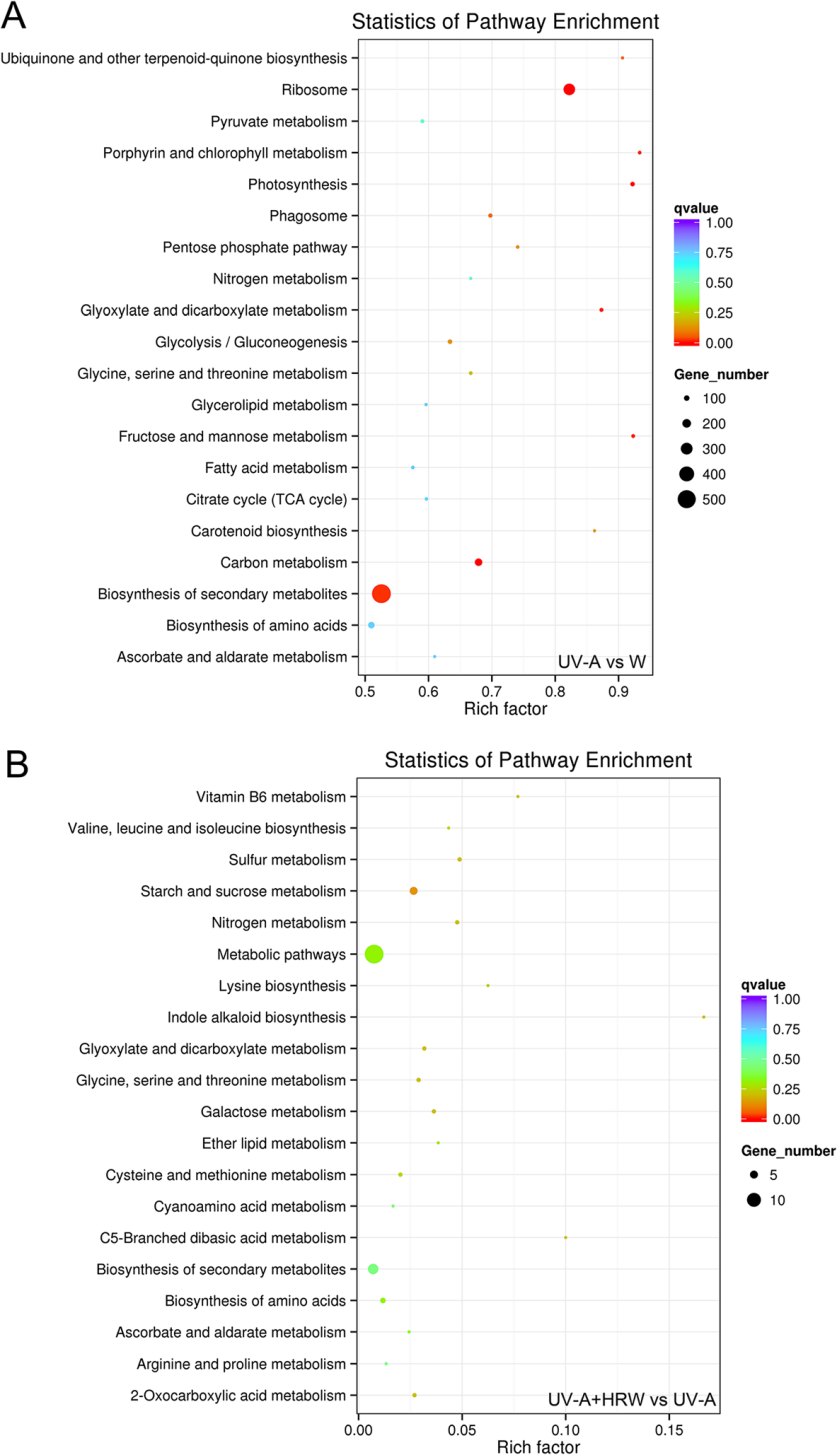
KEGG pathway enrichment analysis of the annotated DEGs. (**a**) UV-A vs. W comparisons; (**b**) UV-A+HRW vs. UV-A comparisons. The Y-axis indicates the KEGG pathway, the X-axis indicates the rich factor. The dot size indicates the number of DEGs of the pathway, and the dot colour indicates the qvalue

To further explore the biological functions of the DEGs, an enrichment analysis based on KEGG database was performed. The top 20 pathways for the most prominent differential genes were listed (Fig. 4). In Fig. 4a, 4504 DEGs were identified in the UV-A vs W comparison, with 2562 up-regulated and 2026 down-regulated. These DEGs were mostly enriched in “ubiquinone and other terpenoid-quinone biosynthesis”, “ribosome”, “porphyrin and chlorophyll metabolism”, “photosynthesis”, “glyoxylate and dicarboxylate metabolism”, “fructose and mannose metabolism”, “carotenoid biosynthesis” and “biosynthesis of secondary metabolites”. While, there were 60 DEGs identified from the KEGG database in the UV-A + HRW vs UV-A comparison, with 29 up-regulated and 31 down-regulated. As shown in Fig. 4b, the DEGs were significantly enriched in “vitamin B6 metabolism”, “sulfur metabolism”, “starch and sucrose metabolism”, “nitrogen metabolism”, “lysine biosynthesis”, “indole alkaloid biosynthesis”, “glyoxylate and dicarboxylate metabolism”, “glycine, serine and threonine metabolism”, “galactose metabolism”, “C5-branched dibasic acid metabolism” and “2-oxocarboxylic acid metabolism”. In particular, “nitrogen metabolism”, “biosynthesis of secondary metabolites”, “phenylpropanoid biosynthesis”, “starch and sucrose metabolism” and “metabolic pathways” were significantly enriched in the KEGG pathway analysis in the UV-A + HRW vs UV-A comparison.

### Expression profiling of structural genes associated with anthocyanin biosynthesis

**Fig. 5:**
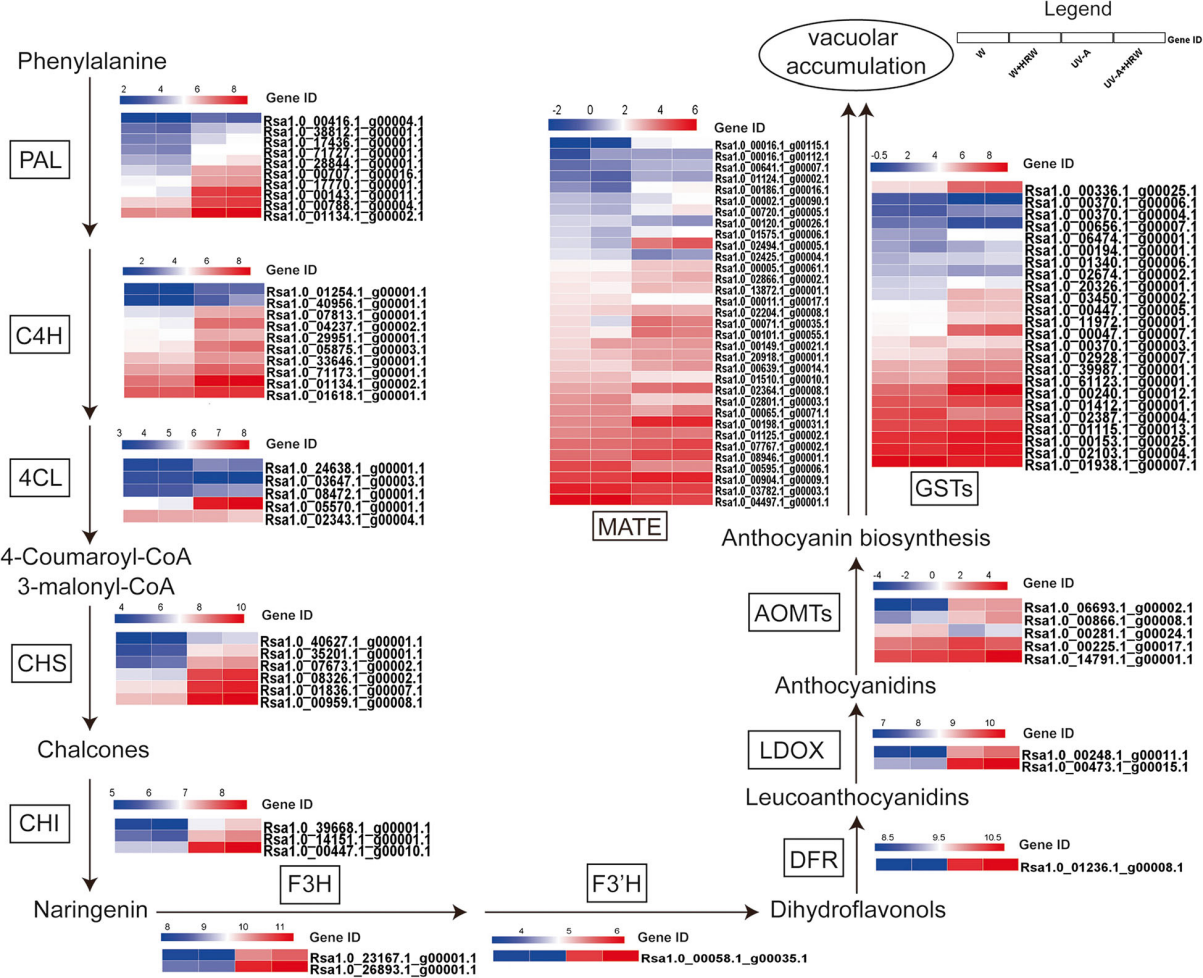
Heat map diagrams of relative expression levels of anthocyanin biosynthesis-related structural genes in response to HRW under W and UV-A radiation in radish sprouts. PAL: phenylalanine ammonia lyase; C4H: Cinnamate 4-hydroxylase; 4CL: 4-coumaroyl: CoA ligase; CHS: chalcone synthase; CHI: chalcone isomerase; F3H: flavanone 3-hydroxylase; F3’H: Flavonoid 3′- hydroxylase; DFR: dihydroflavonol-4-reductase; LDOX: leucoanthocyanidin dioxygenase; OMT: O-methyltransferase; GST: glutathione S-transferase; MATE: Multidrug and Toxic Compound Extrusion (MATE) transporter families. Enzyme names are indicated in the box, and gene IDs are indicated at the right side of each heat map

Anthocyanins are synthesized by the flavonoid pathway, and many enzymes participate in the catalyzing steps. In our current study, the transcript levels of 12 anthocyanin biosynthesis-related structural genes were analyzed (Fig. 5), and the expression levels of most of those genes were higher under UV-A than W. Ten putative *PAL* (*phenylalanine ammonia lyase*) genes were identified, most of which (except for *Rsa1.0_71727.1_g00001.1* and *Rsa1.0_00416.1_g00004.1*), were up-regulated under UV-A + HRW compared with UV-A. Ten *C4H* (*Cinnamate 4-hydroxylase*) genes were identified. The expression levels of *Rsa1.0_05875.1_g00003.1*, *Rsa1.0_07813.1_g00001.1* and *Rsa1.0_40956.1_g00001.1* under UV-A + HRW were higher than those under UV-A. Five *4CL* (*4-coumaroyl:CoA ligase*) genes were identified, among which the expression levels of *Rsa1.0_05570.1_g00001.1* and *Rsa1.0_08472.1_g00001.1* were higher under UV-A + HRW than UV-A. CHS plays an important role in anthocyanin biosynthesis, and six putative *CHS* (*chalcone synthase*) genes identified here displayed similar expression profiles, which were up-regulated under UV-A + HRW compared with UV-A., In addition, three *CHI* (*chalcone isomerase*) genes, two *F3H* (*flavanone 3-hydroxylase*) genes, one *F3’H* (*flavanone 3′-hydroxylase*) gene, one *DFR* (*dihydroflavonol 4-reductase*) gene and two *LDOX* (*leucoanthocyanidin dioxygenase*) genes were identified, and they showed expression patterns similar to those of *CHS*.

In this study, 5 putative *OMT* (*O-methyltransferase*) genes were identified, and the expression levels of *Rsa1.0_00866.1_g00008.1*, *Rsa1.0_06693.1_g00002.1* and *R sa1.0_14791.1_g00001.1* were increased under UV-A and further increased under UV-A + HRW relative to W. We identified 24 putative *GST* (*glutathione S-transferase*)-encoding genes here. Fifteen putative *GST* genes were up-regulated under UV-A + HRW compared with UV-A, including *Rsa1.0_00336.1_g00025.1*, *Rsa1.0_02928.1_g00007.1* and *Rsa1.0_00370.1_g00004.1*. In addition, 33 putative *MATE* genes were identified, and the expression levels of *Rsa1.0_02494.1_g00005.1* and *Rsa1.0_00065.1_g00071.1* were significantly up-regulated under UV-A + HRW compared with UV-A.

### Expression profiling of transcription factors associated with anthocyanin biosynthesis

**Fig. 6:**
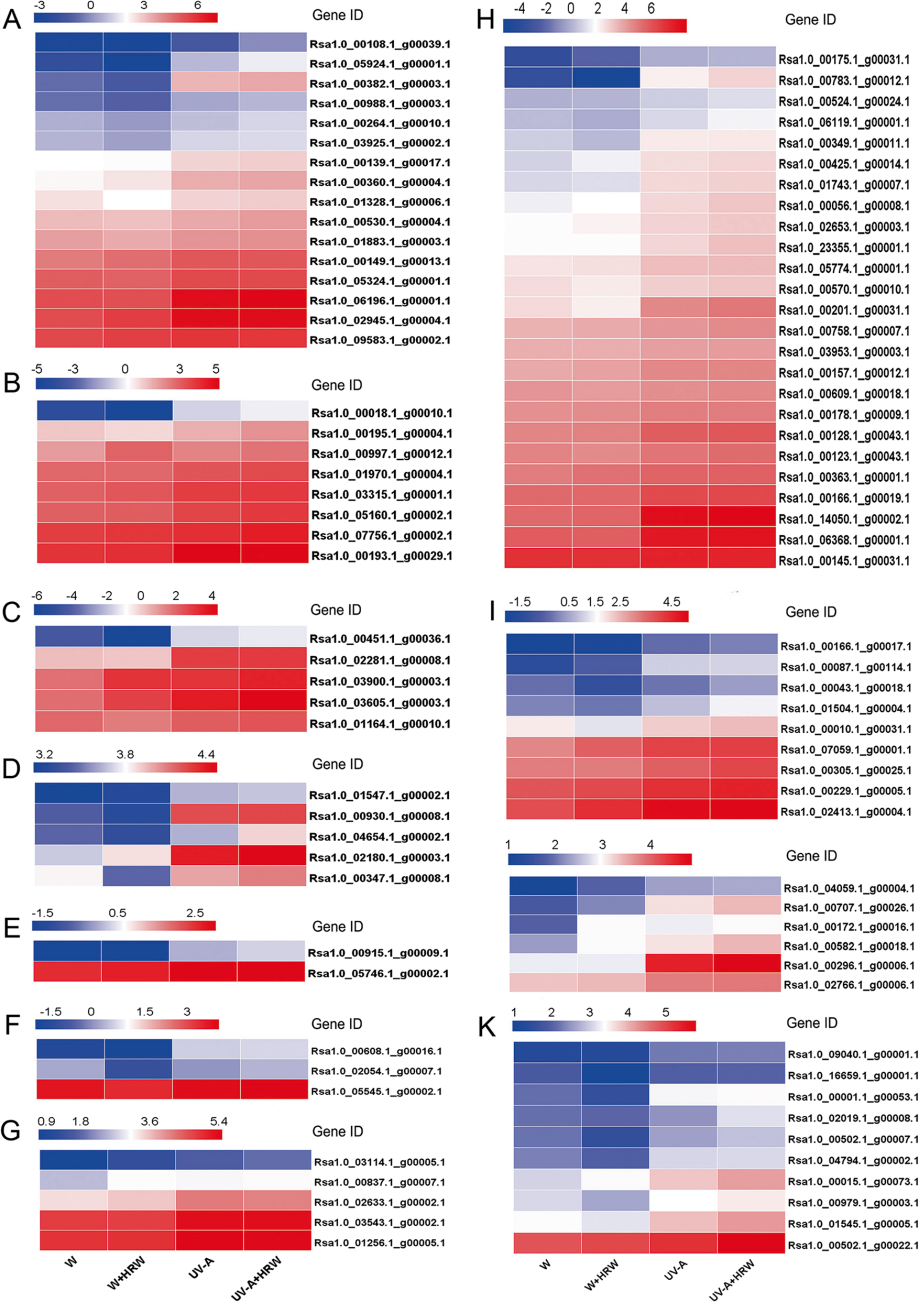
Heat map diagrams of relative expression levels of TFs annotated in anthocyanin biosynthesis in radish sprouts. **a** bHLH, basic helix-loop-helix; (**b**) bZIP, basic region/leucine zipper; (**c**) C2H2, C2H2 zinc-finger proteins; (**d**) C3H, Cys3His zinc finger; (**e**) FAR1, FAR-RED IMPAIRED RESPONSE1; (**f**) TCP, TCP proteins; (**g**) WD40 (WD40 repeat proteins) (**h**) MYB, MYB proteins; (**i**) NAC, NAM/ATAF/CUC; (**j**) WRKY, WRKY proteins; (**k**) zf-HD, zf-HD proteins. Columns in the heat map represent the samples of different treatment. The color scale on the top represents the log-transformed FPKM value

Transcription factors play important regulatory roles in anthocyanin biosynthesis. In this study, a total of 94 transcription factors were identified as putative regulators of anthocyanin biosynthesis in response to HRW. These TFs included bHLHs (basic helix-loop-helix), bZIPs (basic region-leucine zipper), C2H2s (C2H2 zinc-finger proteins), C3Hs (Cys3His zinc finger proteins), FAR1s (far-red impaired response 1), WD40s, MYBs (MYB domain proteins), NACs (NAM/ATAF/CUC), TCPs (TCP proteins), WRKYs (WRKY proteins), and zf-HD proteins (Fig. 6).

Among the various TFs, MYB composed the largest percentage (26.60%), followed by bHLH (17.02%) and WD40 (5.32%), indicating that the MBW complex might be the major determinant of anthocyanin biosynthesis under UV-A + HRW in radish sprouts (Fig. 6 a, g, h). All 16 of the putative bHLH TFs were significantly up-regulated under UV-A treatment compared to W, and UV-A + HRW treatment further promoted this effect (Fig. 6a). In this study, 25 putative MYB genes were identified, and most of them (except *Rsa1.0_00145.1_g00031.1_0*) were up-regulated under UV-A + HRW treatment compared with UV-A treatment (Fig. 6h). Furthermore, 8 putative bZIPs, 10 putative NACs and 9 putative zf-HDs were identified in this study, and their expression levels increased under UV-A + HRW treatment compared to UV-A, implying that these TFs might positively regulate HRW-promoted anthocyanin biosynthesis (Fig. 6b, i, k). In addition, 5 other families (C2H2, C3H, FAR1, TCP and WRKY) showed up-regulation or down-regulation under UV-A + HRW treatment compared to UV-A, implying that these TFs might be involved in HRW-regulated anthocyanin biosynthesis (Fig. 6).

### The differentially expressed genes involved in anthocyanin biosynthesis-related signaling pathways

Light signals are perceived by plants through different classes of photoreceptors. CRY and PHOT mediate blue light/UV-A signal transduction. In our study, based on the KEGG and GO pathway annotations, three putative CRYs were found, and compared to W, their expression levels increased under UV-A but were lower under UV-A + HRW. Among the eight putative PHOT genes, the expression levels of *Rsa1.0_08201.1_g00003.1* and *Rsa1.0_51768.1_g00001.1* increased under UV-A + HRW compared with UV-A, although there was no significant difference. The expression level of a putative COP1 (*Rsa1.0_02834.1_g00003.1*) increased under UV-A compared with W and remained the same under UV-A + HRW. Expression of a putative HY5 (*Rsa1.0_02208.1_g00006.1*) decreased under both UV-A and UV-A + HRW relative to W (Additional file 2: Table S2).

Plant hormones have a crucial role in the regulation of anthocyanin biosynthesis. It has been reported that ABA, JA, cytokinins and GA promote light-induced anthocyanin biosynthesis [45–48]. In our study, eight putative cytokinin-related genes were identified, of which the expression levels of *Rsa1.0_00570.1_g00019.1* and *Rsa1.0_01905.1_g00006.1* increased under UV-A relative to W. The expression level of one putative auxin-related gene (*Rsa1.0_02398.1_g00002.1*) decreased under UV-A + HRW compared with UV-A.

We also investigated the genes of MAPK and calcium signaling pathways. We found that the expression levels of one putative MAPK signaling-related gene (*Rsa1.0_00250.1_g00016.1*) and two calcium signaling-related genes (*Rsa1.0_00024.1_g00018.1* and *Rsa1.0_07482.1_g00001.1*) decreased under UV-A + HRW compared with UV-A. All of these results suggested that light and hormone signals play a vital role in UV-A-induced anthocyanin biosynthesis and might also take part in H_2_-promoted anthocyanin accumulation under UV-A.

### qRT-PCR validation of differentially expressed genes

To validate the RNA-seq data, 16 anthocyanin biosynthesis-related genes were chosen for validation of expression by qRT-PCR analysis (Additional file 3: Figure S1). These 16 genes included those involved in UV-A photoreception and light signal perception, as well as transcription factors and structural genes involved in anthocyanin biosynthesis. In the qRT-PCR analysis, the expression of these genes showed patterns very similar to those of the FPKM values from sequencing under the corresponding treatments. These results indicated that the RNA-seq data were reliable.

## Discussion

Radishes are members of the Brassicaceae, and radish sprouts are important edible vegetables that receive significant attention for their high nutrient contents and health-promoting effects. Anthocyanins, which confer vivid red hypocotyls that draw consumers to radish sprouts, are among these important health-promoting compounds. Therefore, various methods and technologies have been applied to produce radish sprouts rich in anthocyanins [29, 36, 49]. Light acts as an essential stimulus and a safe regulator for anthocyanin biosynthesis. In recent decades, the understanding of the mechanisms underlying light-regulated anthocyanin biosynthesis in plants has markedly increased [50, 51]. Specifically, UV-A could induce anthocyanin accumulation with little damage to plants.

Molecular hydrogen (H_2_) has been identified as a novel antioxidant that can efficiently reduce oxidative stress. The progress toward therapeutic and preventive applications of hydrogen has shown that supplementation with hydrogen-rich water may have a role in preventing of insulin resistance and type 2 diabetes mellitus (T2DM) [52]. More recently, studies on animals showed that HRW could protect against ischemic brain injury in rats, and the maintenance of parvalbumin and hippocalcin levels by HRW might contribute to neuroprotective effects against neuron damage [53]. Therapeutic hydrogen has been administered by different delivery methods, including straightforward inhalation, injection with hydrogen-saturated saline and drinking hydrogen dissolved in water [23]. In plants, external supplementation with H_2_ in the form of HRW protects plants from multiple environmental stimuli and enhances their antioxidant capacity [31, 32, 54]. Recently, it was reported that UV-B-triggered biosynthesis of (iso)flavonoids was substantially strengthened by HRW [34], and the accumulated (iso)flavonoids enhanced the tolerance of alfalfa to UV-B. Based on those cumulative knowledge, the beneficial biological effects of hydrogen have been preliminarily proved. In our recent study, the observation that UV-A-induced anthocyanin biosynthesis was further increased by supplementation with HRW was first reported [36]. Furthermore, HRW significantly blocked UV-A-induced ROS accumulation and enhanced the activities of antioxidant enzymes to reestablish ROS homeostasis. However, to date, there has been no study on the identification of DEGs related to H_2_-promoted anthocyanin biosynthesis under UV-A, and little is known of related signaling pathways. This is the first study to investigate the transcriptional changes associated with anthocyanin accumulation under UV-A + HRW.

The present study showed that the expression of most structural genes associated with anthocyanin biosynthesis was up-regulated by UV-A and UV-A + HRW (Fig. 5). The accumulation of anthocyanin in radish sprouts hypocotyls is a complex biological process that is driven by different regulatory factors, including plant hormones, transcription factors and other signal transduction pathways. In our study, 11 UV-A photoreceptors, including three CRY and eight PHOT, were identified (Additional file 2: Table S2). The transcript levels of most of these photoreceptors increased under UV-A compared with W and did not increase when HRW was applied. Downstream of the light receptors, the ubiquitin E3 ligase CONSTITUTIVE PHOTOMORPHOGENIC1 (COP1) acts as a molecular switch of light-induced plant development, mediating the degradation of various photomorphogenesis-promoting transcription factors by the Ub-proteasome system [13, 55]. ELONGATED HYPOCOTYL5 (HY5) is a bZIP transcription factor that has been linked to activation of *CHS* and the accumulation of flavonoids in response to light in *Arabidopsis* [56]. One COP1 was identified in this study, and its transcript level increased under UV-A. Among the three HY5 found, the transcript levels of two increased under UV-A, and the other decreased. Interestingly, the transcript levels of COP1 and HY5 did not increase under UV-A + HRW compared with UV-A. These results indicated that the photoreceptors COP1 and HY5 might participate in UV-A-induced anthocyanin biosynthesis, but the expression levels of these photoreceptors were not affected by HRW.

Plant hormones may be important factors regulating the light-induced anthocyanin accumulation. For example, auxin has been found to retard anthocyanin accumulation in apple and *Arabidopsis* [57, 58]. In the present study, the transcript level of one auxin-associated gene (*Rsa1.0_02398.1_g00002.1*) decreased (Additional file 2: Table S2), implying that it may participate in anthocyanin biosynthesis under UV-A + HRW. Other plant hormones, such as abscisic acid, cytokinins, and jasmonic acid have been reported to promote anthocyanin accumulation [45, 47, 48]. However, in this study, the transcript levels of most of those hormone-related genes remained unchanged under UV-A + HRW compared with UV-A. These results are not consistent with our expectation. Recently, it was reported that H_2_ could be a novel signaling molecule in phytohormone signaling pathways in response to biotic and abiotic stresses [59]. Thus, further pharmacological experiments will be necessary to verify the role of phytohormones in H_2_-promoted anthocyanin biosynthesis under UV-A. In addition, one transcript encoding a gene involved in MAPK signaling and two transcripts encoding genes involved in calcium signaling were down-regulated under UV-A + HRW. Anthocyanin pigmentation is modulated by changes in intracellular Ca^2+^ level in *Arabidopsis* [60], and application of a CaCl_2_ spray could enhance accumulation of anthocyanins in strawberry [61]. These results imply that Ca^2+^ may take part in H_2_-promoted anthocyanin biosynthesis under UV-A, although additional evidence needs to be obtained.

The molecular basis of plant anthocyanin biosynthesis is well known. Transcription factors regulating the expression of flavonoid biosynthetic genes have been characterized extensively in *Arabidopsis* and other plants [16, 62, 63]. In Arabidopsis, three transcription factor families, including R2R3-MYB, basic helix-loop-helix (bHLH), and WD40-repeat proteins, constitute a ternary complex predominantly responsible for orchestrating anthocyanin biosynthesis [13]. MYB proteins, which are widely distributed in all eukaryotic organisms, constitute one of the largest transcription factor families in the plant kingdom. The production of anthocyanin is predominantly regulated by R2R3-MYB [64]. In this study, we identified 89 unique transcripts encoding members of 10 transcription factor families, which were annotated in anthocyanin biosynthesis in radish sprouts (Fig. 6). Among those TFs, 25 putative MYB-encoding genes, 16 putative bHLH-encoding genes and 2 putative WD40-encoding genes were identified. Those three TFs accounted for approximately one-half of the total number of TFs identified. This finding suggested that the MBW complex is likely to be the primary regulator of anthocyanin accumulation in radish sprouts under UV-A + HRW treatment. In peach, PpNAC1 can activate the transcription of *PpMYB10.1* to promote anthocyanin pigmentation [65]. In addition, it has been suggested that IbNAC1 has multiple biological functions in the JA response, including the inhibition of root formation and accumulation of anthocyanin in sweet potato [66]. In our present study, 9 NACs identified in radish sprouts showed increased expression levels under UV-A + HRW treatment. In addition, the co-up-regulation of NAC and MYB implied that the activation of MYB might be associated with NAC. In our study, the expression levels of most transcription factors annotated in anthocyanin biosynthesis increased under UV-A and were further enhanced by HRW under UV-A. These findings showed that the regulatory effects of TFs might play vital roles in H_2_-promoted anthocyanin biosynthesis under UV-A.

In this study, the H_2_ production of radish sprouts was significantly increased under UV-A (Fig. 1c). Similarly, the UV-B irradiation was reported to significantly increase the H_2_ content in alfalfa [34]. We observed that endogenous H_2_ content increased under abiotic stress in higher plants, including alfalfa [34], Chinese cabbage [32] and *Arabidopsis* [31]. In bacteria and green algae, a hydrogenase is responsible for H_2_ production [67]. However, no reports have clarified the mechanism of H_2_ production in higher plants. With current levels of technology, it is difficult to answer these questions completely. In future, these problems will gradually be solved with improved scientific methods and increased attention to the physiological role of H_2_ in higher plants.

**Fig. 7:**
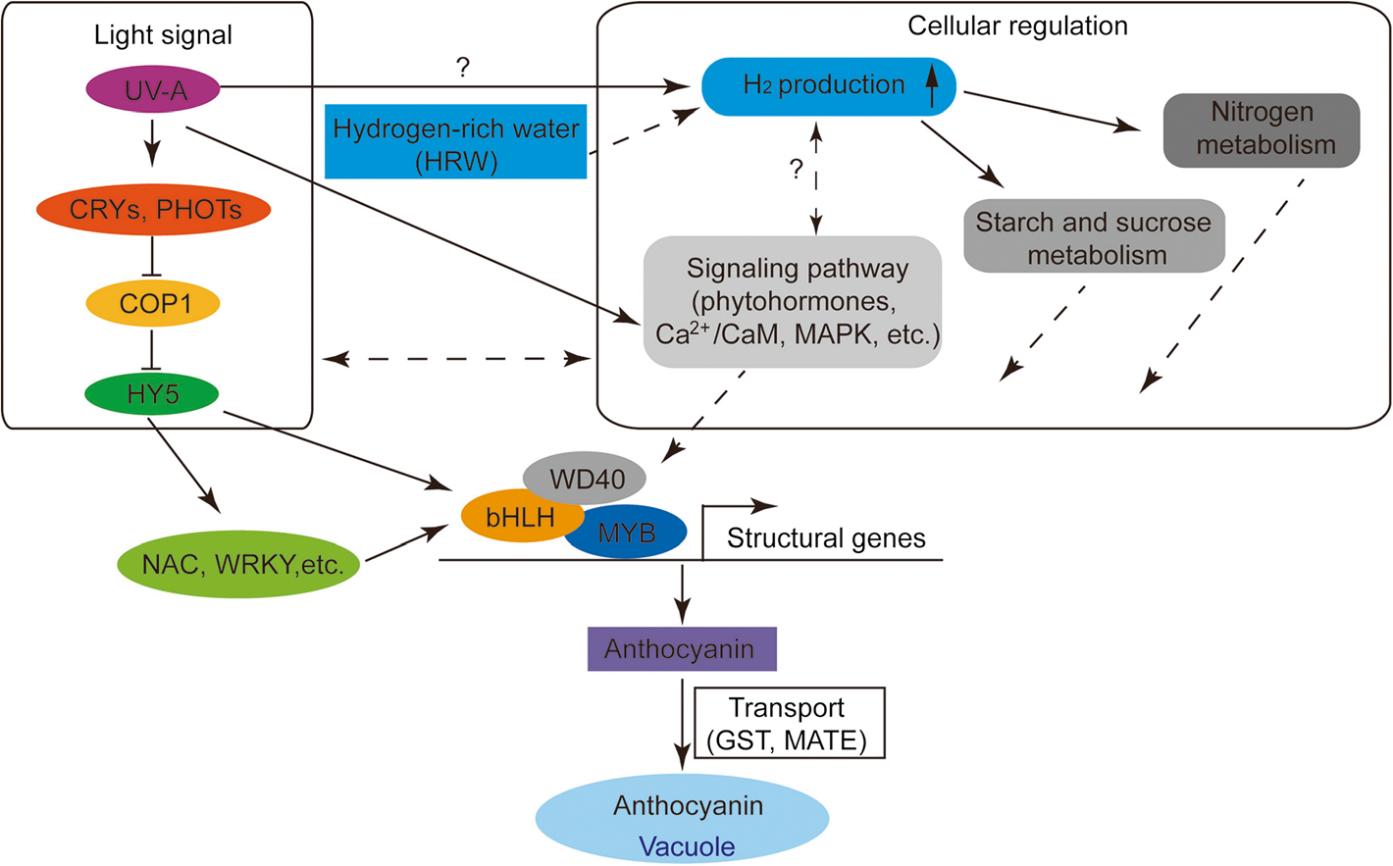
A proposed model of HRW-promoted anthocyanin biosynthesis under UV-A radiation

Based on this transcriptomic analysis and previous studies, a putative regulatory network of HRW-promoted anthocyanin accumulation in radish sprouts under UV-A radiation was proposed (Fig. 7). Under UV-A radiation, UV-A photoreceptors, such as CRYs and PHOTs, perceive light signals and then interact with COP1, which interacts directly with positive regulators of photomorphogenesis, such as HY5. HY5 can interact with MBW complex or other transcription factors to regulate the transcription of structural genes. On the other hand, UV-A promotes the production of H_2_, which promote signaling pathways (such as phytohormones, Ca^2+^/CaM and MAPK), sugar metabolism and nitrogen metabolism to regulate anthocyanin biosynthesis. Supplementation of HRW significantly increased the H_2_ concentration. This could at least partially explain why anthocyanin accumulation was substantially improved by HRW under UV-A. Further physiological and molecular experiments should be conducted to provide more precise insight into the mechanisms of HRW-promoted anthocyanin biosynthesis under UV-A, as well as the production of endogenous H_2_.

## Conclusion

Four sets of transcriptome data comprising 80,522 unigenes were identified in radish sprouts by Illumina sequencing. A total of 14,564 DEGs were identified through pairwise comparisons. GO and KEGG pathway enrichment analyses revealed the DEGs involved in HRW-promoted anthocyanin biosynthesis under UV-A. Genes encoding light signal transduction elements, anthocyanin biosynthesis-related TFs and structural genes, and anthocyanin biosynthesis-related signaling pathways were also identified. The transcriptome data provided valuable information and gene sequences to accelerate the process of revealing the regulatory mechanism of HRW-promoted anthocyanin biosynthesis under UV-A in radish sprouts. In addition, further studies are needed to confirm the precise mechanisms by which H_2_ regulates physiological processes.

## Additional files

Additional file 1: **Table S1.** The primers of 16 DEGs validated by qRT-PCR analysis. (XLS 33 kb)

Additional file 2: **Table S2.** The differentially expressed genes involved in anthocyanin biosynthesis-related signaling pathways. (XLS 131 kb)

Additional file 3: **Figure S1.** Expression of anthocyanin biosynthesis-related genes quantified by RNA-seq and qRT-PCR analysis. The left y-axis indicates the log2 of FPKM values of genes from RNA-seq data (black columns). The right y-axis indicates relative gene expression levels analyzed by qRT-PCR (white columns), values are presented as mean ± SE (*n* = 3). (TIF 217 kb)

## Abbreviations

4CL: 4-coumaroyl:CoA ligase
ANS: anthocyanidin synthase
C4H: Cinnamate 4-hydroxylase
CHI: chalcone isomerase
CHS: chalcone synthase
DEGs: differentially expressed genes
DFR: dihydroflavonol 4-reductase
EBGs: early biosynthetic genes
F3’H: flavanone 3′-hydroxylase
F3H: flavanone 3-hydroxylase
GC: gas chromatography
GO: Gene Ontology
GST: glutathione S-transferase
H_2_: Hydrogen gas
HRW: hydrogen-rich water
KEGG: Kyoto Encyclopedia of Genes and Genomes
LBGs: late biosynthetic genes
LDOX: leucoanthocyanidin dioxygenase
MBW: MYB-bHLH-WD40
OMT: O-methyltransferase
PAL: phenylalanine ammonia lyase
qPCR: real time quantitative PCR
RNA-seq: RNA-sequencing
ROS: Reactive oxygen species
UFGT: UDP glucose:flavonoid 3-O-glucosyltransferase
UV-A: UV-A radiation
W: white light

## References

[1] Yuan G, Wang X, Guo R, Wang Q (2010). Effect of salt stress on phenolic compounds, glucosinolates, myrosinase and antioxidant activity in radish sprouts. Food Chem.

[2] Zhou C, Zhu Y, Luo Y (2013). Effects of sulfur fertilization on the accumulation of health-promoting phytochemicals in radish sprouts. J Agric Food Chem.

[3] Holton TA, Cornish EC (1995). Genetics and biochemistry of anthocyanin biosynthesis. Plant Cell.

[4] Hatier JHB, Gould KS (2008). Anthocyanin function in vegetative organs. Anthocyanins.

[5] Cutuli B, Lemanski C, Fourquet A, Lafontan BD, Giard S, Lancrenon S, Meunier A, Pioud-Martigny R, Campana F, Marsiglia H (2010). Environmental significance of anthocyanins in plant stress responses. Photochem Photobiol.

[6] Pascualteresa SD, Moreno DA, Garcíaviguera C (2009). Flavanols and anthocyanins in cardiovascular health: a review of current evidence. Int J Mol Sci.

[7] Wang H, Nair MG, Strasburg GM, Chang YC, Booren AM, Gray JI, Dewitt DL (1999). Antioxidant and antiinflammatory activities of anthocyanins and their aglycon, cyanidin, from tart cherries. J Nat Prod.

[8] Wang LS, Stoner GD (2008). Anthocyanins and their role in cancer prevention. Cancer Lett.

[9] Gonzalez A, Zhao M, Leavitt JM, Lloyd AM (2008). Regulation of the anthocyanin biosynthetic pathway by the TTG1/bHLH/Myb transcriptional complex in Arabidopsis seedlings. Plant J.

[10] Jaakola L, Määttä K, Pirttilä AM, Törrönen R, Kärenlampi S, Hohtola A (2002). Expression of genes involved in anthocyanin biosynthesis in relation to anthocyanin, proanthocyanidin, and flavonol levels during bilberry fruit development. Plant Physiol.

[11] Broun P (2005). Transcriptional control of flavonoid biosynthesis: a complex network of conserved regulators involved in multiple aspects of differentiation in *Arabidopsis*. Curr Opin Plant Biol.

[12] Hichri I, Barrieu F, Bogs J, Kappel C, Delrot S, Lauvergeat V (2011). Recent advances in the transcriptional regulation of the flavonoid biosynthetic pathway. J Exp Bot.

[13] Jaakola L (2013). New insights into the regulation of anthocyanin biosynthesis in fruits. Trends Plant Sci.

[14] Jeong ST, Goto-Yamamoto N, Kobayashi S, Esaka M (2004). Effects of plant hormones and shading on the accumulation of anthocyanins and the expression of anthocyanin biosynthetic genes in grape berry skins. Plant Sci.

[15] Loreti E, Povero G, Novi G, Solfanelli C, Alpi A, Perata P (2008). Gibberellins, jasmonate and abscisic acid modulate the sucrose-induced expression of anthocyanin biosynthetic genes in *Arabidopsis*. New Phytol.

[16] Lotkowska ME, Tohge T, Fernie AR, Xue GP, Balazadeh S, Muellerroeber B (2015). The Arabidopsis transcription factor MYB112 promotes anthocyanin formation during salinity and under high light stress. Plant Physiol.

[17] Christie JM, Jenkins GI (1996). Distinct UV-B and UV-A/blue light signal transduction pathways induce chalcone synthase gene expression in *Arabidopsis* cells. Plant Cell.

[18] Wang Y, Zhou B, Sun M, Li Y, Kawabata S (2012). UV-A light induces anthocyanin biosynthesis in a manner distinct from synergistic blue+UV-B light and UV-A/blue light responses in different parts of the hypocotyls in turnip seedlings. Plant Cell Physiol.

[19] Zhou B, Li Y, Xu Z, Yan H, Homma S, Kawabata S (2007). Ultraviolet A-specific induction of anthocyanin biosynthesis in the swollen hypocotyls of turnip (*Brassica rapa*). J Exp Bot.

[20] Jia G, Wang MH (2010). Ultraviolet A-specific induction of anthocyanin biosynthesis and PAL expression in tomato (*Solanum lycopersicum* L.). Plant Growth Regul.

[21] Nguyen CT, Kim J, Yoo KS, Lim S, Lee EJ (2014). Effect of prestorage UV-A, -B, and -C radiation on fruit quality and anthocyanin of 'Duke' blueberries during cold storage. J Agric Food Chem.

[22] Hong Y, Chen S, Zhang JM (2010). Hydrogen as a selective antioxidant: a review of clinical and experimental studies. J Int Med Res.

[23] Huang CS, Kawamura T, Toyoda Y, Nakao A (2010). Recent advances in hydrogen research as a therapeutic medical gas. Free Radic Res.

[24] Ohsawa I, Ishikawa M, Takahashi K, Watanabe M, Nishimaki K, Yamagata K, Katsura K, Katayama Y, Asoh S, Ohta S (2007). Hydrogen acts as a therapeutic antioxidant by selectively reducing cytotoxic oxygen radicals. Nat Med.

[25] Ohta S (2011). Recent progress toward hydrogen medicine: potential of molecular hydrogen for preventive and therapeutic applications. Cur Pharm Des.

[26] Shen M, Zhang H, Yu C, Wang F, Sun X (2014). A review of experimental studies of hydrogen as a new therapeutic agent in emergency and critical care medicine. Med Gas Res.

[27] Liu C, Cui J, Sun Q, Cai J (2010). Hydrogen therapy may be an effective and specific novel treatment for acute radiation syndrome. Med Hypotheses.

[28] Jin Q, Zhu K, Cui W, Xie Y, Han B, Shen W (2013). Hydrogen gas acts as a novel bioactive molecule in enhancing plant tolerance to paraquat-induced oxidative stress via the modulation of heme oxygenase-1 signalling system. Plant Cell Environ.

[29] Wu Q, Su N, Zhang X, Liu Y, Cui J, Liang Y (2016). Hydrogen peroxide, nitric oxide and UV RESISTANCE LOCUS8 interact to mediate UV-B-induced anthocyanin biosynthesis in radish sprouts. Sci Rep.

[30] Xie Y, Mao Y, Lai D, Zhang W, Shen W (2012). H_2_ enhances *Arabidopsis* salt tolerance by manipulating ZAT10/12-mediated antioxidant defence and controlling sodium exclusion. PLoS One.

[31] Xie Y, Mao Y, Zhang W, Lai D, Wang Q, Shen W (2014). Reactive oxygen species-dependent nitric oxide production contributes to hydrogen-promoted stomatal closure in *Arabidopsis*. Plant Physiol.

[32] Wu Q, Su N, Chen Q, Shen W, Shen Z, Xia Y, Cui J (2015). Cadmium-induced hydrogen accumulation is involved in cadmium tolerance in *Brassica campestris* by reestablishment of reduced glutathione homeostasis. PLoS One.

[33] Xu S, Zhu S, Jiang Y, Wang N, Wang R, Shen W, Yang J (2013). Hydrogen-rich water alleviates salt stress in rice during seed germination. Plant Soil.

[34] Xie Y, Zhang W, Duan X, Dai C, Zhang Y, Cui W, Wang R, Shen W (2015). Hydrogen-rich water-alleviated ultraviolet-B-triggered oxidative damage is partially associated with the manipulation of the metabolism of (iso)flavonoids and antioxidant defence in *Medicago sativa*. Funct Plant Biol.

[35] Zhu Y, Liao W, Wang M, Niu L, Xu Q, Jin X (2016). Nitric oxide is required for hydrogen gas-induced adventitious root formation in cucumber. J Plant Physiol.

[36] Su N, Wu Q, Liu Y, Cai J, Shen W, Xia K, Cui J (2014). Hydrogen-rich water reestablishes ROS homeostasis but exerts differential effects on anthocyanin synthesis in two varieties of radish sprouts under UV-A irradiation. J Agric Food Chem.

[37] Ye X, Tan H, Ma Z, Huang J (2016). DELLA proteins promote anthocyanin biosynthesis via sequestering MYBL2 and JAZ suppressors of the MYB/bHLH/WD40 complex in *Arabidopsis thaliana*. Mol Plant.

[38] Trapnell C, Pachter L, Salzberg SL (2009). TopHat: discovering splice junctions with RNA-Seq. Bioinformatics.

[39] Anders S, Huber W (2012). Differential expression of RNA-Seq data at the gene level–the DESeq package.

[40] Anders S, Huber W (2010). Differential expression analysis for sequence count data. Genome Biol.

[41] Young MD, Wakefield MJ, Smyth GK, Oshlack A (2010). Gene ontology analysis for RNA-seq: accounting for selection bias. Genome Biol.

[42] Xu Y, Zhu X, Gong Y, Xu L, Wang Y, Liu L (2012). Evaluation of reference genes for gene expression studies in radish (*Raphanus sativus* L.) using quantitative real-time PCR. Biochem Biophys Res Commun.

[43] Vandesompele Jo, De Preter Katleen, Pattyn Filip, Poppe Bruce, Van Roy Nadine, De Paepe Anne, Speleman Frank (2002). Genome Biology.

[44] Livak KJ, Schmittgen TD (2012). Analysis of relative gene expression data using real-time quantitative PCR and the 2^−ΔΔCT^ method. Methods.

[45] Jia HF, Chai YM, Li CL, Lu D, Luo JJ, Qin L, Shen YY (2011). Abscisic acid plays an important role in the regulation of strawberry fruit ripening. Plant Physiol.

[46] Awad MA, Jager AD (2002). Formation of flavonoids, especially anthocyanin and chlorogenic acid in 'Jonagold' apple skin: infuences of growth regulators and fruit maturity. Scien Horticul.

[47] Das PK, Dong HS, Choi SB, Yoo SD, Choi G, Park YI (2012). Cytokinins enhance sugar-induced anthocyanin biosynthesis in Arabidopsis. Mol Cells.

[48] Shan X, Zhang Y, Peng W, Wang Z, Xie D (2009). Molecular mechanism for jasmonate-induction of anthocyanin accumulation in *Arabidopsis*. J Exp Bot.

[49] Park WT, Kim YB, Seo JM, Kim SJ, Chung E, Lee JH, Sang UP (2013). Accumulation of anthocyanin and associated gene expression in radish sprouts exposed to light and methyl jasmonate. J Agric Food Chem.

[50] Li YY, Mao K, Zhao C, Zhao XY, Zhang HL, Shu HR, Hao YJ (2012). MdCOP1 ubiquitin E3 ligases interact with MdMYB1 to regulate light-induced anthocyanin biosynthesis and red fruit coloration in apple. Plant Physiol.

[51] Jiang M, Ren L, Lian H, Liu Y, Chen H (2016). Novel insight into the mechanism underlying light-controlled anthocyanin accumulation in eggplant (*Solanum melongena* L.). Plant Sci.

[52] Kajiyama S, Hasegawa G, Asano M, Hosoda H, Fukui M, Nakamura N, Kitawaki J, Imai S, Nakano K, Ohta M (2008). Supplementation of hydrogen-rich water improves lipid and glucose metabolism in patients with type 2 diabetes or impaired glucose tolerance. Nutr Res.

[53] Han L, Tian R, Yan H, Pei L, Hou Z, Hao S, Li YV, Tian Q, Liu B, Zhang Q (2015). Hydrogen-rich water protects against ischemic brain injury in rats by regulating calcium buffering proteins. Brain Res.

[54] Cui W, Fang P, Zhu K, Mao Y, Gao C, Xie Y, Wang J, Shen W (2014). Hydrogen-rich water confers plant tolerance to mercury toxicity in alfalfa seedlings. Ecotoxico Environ Saf.

[55] Liu H, Liu B, Zhao C, Pepper M, Lin C (2011). The action mechanisms of plant cryptochromes. Trends Plant Sci.

[56] Stracke R, Favory JJ, Gruber H, Bartelniewoehner L, Bartels S, Binkert M, Funk M, Weisshaar B, Ulm R (2010). The Arabidopsis bZIP transcription factor HY5 regulates expression of the PFG1/MYB12 gene in response to light and ultraviolet-B radiation. Plant Cell Environ.

[57] Ji XH, Zhang R, Wang N, Yang L, Chen XS (2015). Transcriptome profiling reveals auxin suppressed anthocyanin biosynthesis in red-fleshed apple callus (*Malus sieversii f . niedzwetzkyana*). Plant Cell Tiss Org(PCTOC).

[58] Liu Z, Shi MZ, Xie DY (2014). Regulation of anthocyanin biosynthesis in Arabidopsis thaliana red pap1-D cells metabolically programmed by auxins. Planta.

[59] Liu F, Li J, Liu Y (2016). Molecular hydrogen can take part in phytohormone signal pathways in wild rice. Biolo Plant.

[60] Dong HS, Choi MG, Lee HK, Cho M, Choi SB, Choi G, Park YI (2013). Calcium dependent sucrose uptake links sugar signaling to anthocyanin biosynthesis in Arabidopsis. Biochem Biophys Res Commun.

[61] Xu W, Peng H, Yang T, Whitaker B, Huang L, Sun J, Chen P (2014). Effect of calcium on strawberry fruit flavonoid pathway gene expression and anthocyanin accumulation. Plant Physiol Biochem.

[62] Hu DG, Sun CH, Ma QJ, You CX, Cheng L, Hao YJ (2016). MdMYB1 regulates anthocyanin and malate accumulation by directly facilitating their transport into vacuoles in apples. Plant Physiol.

[63] Lai B, Du LN, Liu R, Hu B, Su WB, Qin YH, Zhao JT, Wang HC, Hu GB (2016). Two LcbHLH transcription factors interacting with LcMYB1 in regulating late structural genes of anthocyanin biosynthesis in *Nicotiana* and *Litchi chinensis* during anthocyanin accumulation. Front Plant Sci.

[64] Stracke R, Holtgräwe D, Schneider J, Pucker B, Sörensen TR, Weisshaar B (2014). Genome-wide identification and characterisation of R2R3-MYB genes in sugar beet (*Beta vulgaris*). BMC Plant Biol.

[65] Zhou H, Linwang K, Wang H, Gu C, Dare AP, Espley RV, He H, Allan AC, Han Y (2015). Molecular genetics of blood-fleshed peach reveals activation of anthocyanin biosynthesis by NAC transcription factors. Plant J.

[66] Chen SP, Lin IW, Chen X, Huang YH, Chang HC, Lo HS, Lu HH, Yeh KW (2016). Sweet potato NAC transcription factor, IbNAC1, up-regulates sporamin gene expression by binding the SWRE motif against mechanical wounding and herbivore attack. Plant J.

[67] Morimoto K, Kimura T, Sakka K, Ohmiya K (2005). Overexpression of a hydrogenase gene in *Clostridium paraputrificum* to enhance hydrogen gas production. FEMS Microbiol Lett.

